# Draft genome of *Rhodotorula mucilaginosa* AIRLRB09-2, an efficient polyurethane degrading yeast isolated from waste gas-treated airlift

**DOI:** 10.1128/mra.01201-25

**Published:** 2026-08-31

**Authors:** Jiří Bárta, Jiří Mach, Martin Halecký

**Affiliations:** 1 Faculty of Science, Centre for Polar Ecology, University of South Bohemia in Ceske Budejovice 204738, České Budějovice, Czechia; 2 Department of Biotechnology, Faculty of Food and biochemical technology, University of Chemistry in Prague 678072, Prague, Czechia; University of California Riverside, Riverside, California, USA

**Keywords:** plastic, biodegradation, fungi, DNA sequencing, biotechnology

## Abstract

The *Rhodotorula mucilaginosa* strain AIRLRB09-2 is a laboratory isolate with proven capability of polyurethane and volatile organic compounds (e.g., acetone and styrene, degradation). Here, we report the draft genome sequence of this yeast isolated from airlift, the bioreactor for waste gas treatment containing acetone and styrene.

## ANNOUNCEMENT

Although bacterial and fungal degradation of polymers has previously been widely studied (1, 2), there are limited numbers of studies on yeast polymer degradation. The availability of draft genome of *Rhodotorula mucilaginosa* strain AIRLRB09-2 will advance research on this species to better explore its potential as a biological degrader of variety plastics, namely, polyurethanes, both in natural environments and as potential substrates for biological recycling and/or reuse with sustainable concepts Cradle to Grave or Cradle to Cradle. The *R. mucilaginosa* strain AIRLRB09-2 was isolated from a mixed culture of airlift treating acetone and styrene waste gases (Czech Republic, Prague; 50°6′12.104″N, 14°23′23.568″E) using Rose-Bengal chloramphenicol agar base CM 549 with a selective supplement SR 78 selective agar (Oxoid, England) at a temperature of bioreactor operation of 20°C. Suspension culture (1 L Erlenmeyer flask, RPM of 200 at a temperature of 25°C) in basic salt medium with glucose (5 g/L) lasting 2 days was used for biomass acquisition for DNA extraction. The basic salt medium with pH 7 had the following composition (g/L): K_2_HPO_4_ 3.6; KH_2_PO_4_ 1.7; (NH_4_)_2_SO_4_ 1,3; MgCl_2_ 0.2 × 10^−3^; ZnSO_4_ × 7H_2_O 6 × 10^−5^; MnSO_4_ × H_2_O 2 × 10^−5^; CuSO_4_ × 5H_2_O 2 × 10^−5^; FeSO_4_ × 7H_2_O 3.2 × 10^−5^; CaSO_4_ × 0.5H_2_O 3 × 10^−5^; CoSO_4_ × 7H_2_O 2 × 10^−5^; Na_2_B_4_O_7_ × 10H_2_O 2 × 10^−6^; and Na_2_MoO_4_ × 2H_2_O 2 × 10^−5^.

For the whole-genome sequencing, fungal DNA was isolated from a pure liquid fungal culture. Following centrifugation (10 min at 10,000 rpm) and removal of the supernatant, approximately 5 g of fungal biomass was aseptically transferred to a precooled sterile mortar. The biomass was frozen with liquid nitrogen and ground to a fine powder using a sterile pestle. Approximately 2.5 g was transferred to extraction buffer, and DNA was extracted following the protocol of the DNeasy PowerLyzer Microbial Kit (Qiagen, USA). The quality of DNA was confirmed by agarose gel electrophoresis, and concentration was determined fluorometrically by Qubit (Thermo Fisher Scientific, UK). The purified DNA extract was sent to SEQme company for library preparation and whole-genome sequencing. Library preparation was done using NEBNext Ultra II DNA Library Prep Kit for Illumina NEBNext Multiplex Oligos for Illumina (Unique Dual Index Primer Pairs). Library QC was confirmed by Agilent Bioanalyzer 2100 High-Sensitivity DNA Kit, Invitrogen Collibri Library Quantification Kit, and Qubit 1X dsDNA High-Sensitivity Assay Kit. Sequencing platform was NovaSeq X Plus—2 × 150 bp. In total, 53,485,444 paired-end reads were obtained. Raw fastq reads were *de novo* assembled by SPAdes v3.15.3 (3) with default parameters. The basic statistics are shown in Table 1. The genome size of *R. mucilaginosa* strain AIRLRB09-2 was estimated to be 20,132,545 bp with 60.51% guanine and cytosine (GC) (Table 1).

**TABLE 1:** QUAST summary of *R. mucilaginosa* strain AIRLRB09-2 genome assembly and annotation (4)^*a*^

Parameter	Genome assembly
Contigs	
Contigs (≥0 bp)	224
Contigs (≥1,000 bp)	153
Contigs (≥10,000 bp)	107
Contigs (≥100,000 bp)	64
Contigs (≥1,000,000 bp)	0
Largest contig	791,116
Total length	
Total length (≥0 bp)	20,132,545
Total length (≥1,000 bp)	20,083,772
Total length (≥10,000 bp)	19,932,022
Total length (≥100,000 bp)	17,957,505
Total length (≥1,000,000 bp)	0
N50	316,997
N75	176,827
L50	23
L75	43
GC (%)	60.51
Average coverage	346.36

^*a*^ All statistics are based on contigs of size ≥500 bp, unless otherwise noted (e.g., “Contigs (≥0 bp)” and “Total length (≥0 bp)” include all contigs).

The completeness of the genome was determined by BUSCO v. 5.8.0 (5). Genome assembly completeness was evaluated using BUSCO. Out of 1,764 BUSCO groups searched, 1,721 were identified as complete, corresponding to 97.6% completeness. Most complete BUSCOs were single-copy genes, with 1,719 complete and single-copy BUSCOs detected (97.4%), whereas only two BUSCOs were duplicated (0.1%). Eleven BUSCOs were fragmented (0.6%), and 32 were missing (1.8%). The final assembly comprised 220 scaffolds and 245 contigs with a total length of 20.09 Mb and a scaffold N50 of 316 kb. Although 76 complete BUSCOs contained internal stop codons, the overall BUSCO profile indicates a highly complete genome assembly with low duplication and limited fragmentation.

## Data Availability

The raw reads were deposited in the Sequence Read archive (SRA) under the Bioproject ID PRJNA1327974, assembled genome under the BioSample ID SAMN52481760, and SRA record accession SRR35794965. The genome assembly of *R. mucilaginosa* strain AIRLRB09-2 accession number is JBSSUU000000000.
